# PET Neuroimaging of Alzheimer's Disease: Radiotracers and Their Utility in Clinical Research

**DOI:** 10.3389/fnagi.2021.624330

**Published:** 2021-05-06

**Authors:** Weiqi Bao, Fang Xie, Chuantao Zuo, Yihui Guan, Yiyun Henry Huang

**Affiliations:** ^1^PET Center, Huanshan Hospital, Fudan University, Shanghai, China; ^2^Department of Radiology and Biomedical Imaging, PET Center, Yale University School of Medicine, New Haven, CT, United States

**Keywords:** PET, neuroimaging, Alzheimer's disease, radiotracer, clinical research

## Abstract

Alzheimer's Disease (AD), the leading cause of senile dementia, is a progressive neurodegenerative disorder affecting millions of people worldwide and exerting tremendous socioeconomic burden on all societies. Although definitive diagnosis of AD is often made in the presence of clinical manifestations in late stages, it is now universally believed that AD is a continuum of disease commencing from the preclinical stage with typical neuropathological alterations appearing decades prior to its first symptom, to the prodromal stage with slight symptoms of amnesia (amnestic mild cognitive impairment, aMCI), and then to the terminal stage with extensive loss of basic cognitive functions, i.e., AD-dementia. Positron emission tomography (PET) radiotracers have been developed in a search to meet the increasing clinical need of early detection and treatment monitoring for AD, with reference to the pathophysiological targets in Alzheimer's brain. These include the pathological aggregations of misfolded proteins such as β-amyloid (Aβ) plagues and neurofibrillary tangles (NFTs), impaired neurotransmitter system, neuroinflammation, as well as deficient synaptic vesicles and glucose utilization. In this article we survey the various PET radiotracers available for AD imaging and discuss their clinical applications especially in terms of early detection and cognitive relevance.

## Introduction

Alzheimer's Disease (AD), the leading cause of senile dementia, is a progressive neurodegenerative disorder affecting millions of people worldwide and exerting tremendous socioeconomic burden on all societies (Goedert and Spillantini, 2006; Querfurth and LaFerla, 2010). AD is neuropathologically characterized by deposition of senile plaques and neurofibrillary tangles in the brain tissue. Excessive aggregation of misfolded β-amyloid (Aβ) and hyperphosphorylated tau proteins leads to cytotoxicity and disruption of cytoarchitecture, and subsequent neuronal death and brain function decline. Neuroinflammation activation, cholinergic deficit, impaired glucose utilization and synaptic dysfunction are also outstanding characteristics of AD. Functional neuroimaging using positron emission tomography (PET) is able to reveal these *in vivo* pathological/pathophysiological alterations. With increasing viewpoint of AD as a continuum from asymptomatic preclinical stage, to prodromal stage with mild cognitive impairment (MCI), and finally to the advanced stage of dementia, integrated early diagnostic and differentiation paradigms with the help of PET imaging has been well-acknowledged in multiple diagnostic criteria (Dubois et al., 2007, 2014; Jack et al., 2011). In this review article, we describe the various PET radiotracers available for AD imaging and discuss their clinical applications especially in terms of early detection and cognitive relevance. Literature evidence on the predictive ability of PET imaging with various PET tracers for prodromal stage conversion and monitoring of disease progression will also be reviewed. Finally, comments on emerging biomarkers and their prospects in early detection of AD will be provided.

## PET Tracers for Imaging Aβ

### Overview

The disturbance of homeostasis between the accumulation of neurotoxic Aβ peptides and its clearance in the brain is believed to be the core event in AD etiology (Hardy and Allsop, 1991; Hardy and Higgins, 1992). The Aβ peptides are derived from the breakdown of amyloid precursor protein (APP) through cleavage by β- and γ-secretase. The soluble oligomers, believed to be the perpetrator of cytotoxicity, are aggregated by longer species of the Aβ peptides such as Aβ_40_ and Aβ_42_ released into the extracellular space. The accumulation of Aβ peptides, from neurotoxic oligomers to further aggregated insoluble β-sheet fibrils and dense fibrillary plaques, is believed to underlie subsequent neurofibrillary tangle formation and neuronal loss, which precede the onset of clinical symptoms by more than 10–15 years (Hardy and Gwinn-Hardy, 1998; Hardy et al., 1998). However, confirmation of AD neuropathology has long relied on immunohistochemical staining of Aβ aggregates in postmortem autopsy tissues. Since the early 2000s, the availability of antemortem *in vivo* PET imaging with Aβ radiotracers has greatly advanced our knowledge on the time course and correlation of Aβ aggregation, AD progression, and cognitive decline, and revolutionized AD diagnosis.

### Tracer Development

**Figure 1:**
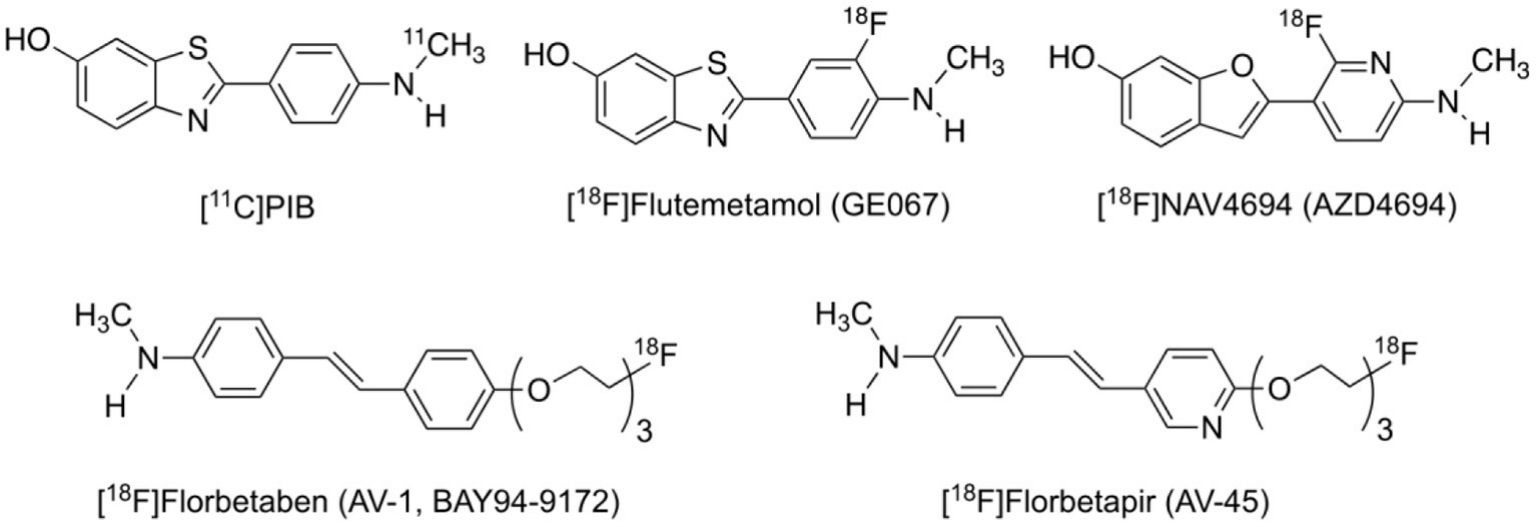
Structures of representative PET radiotracers for Aβ imaging.

The search for Aβ imaging tracers dated back to the mid-1990s, culminated in the first *in vivo* imaging of Aβ in an AD patient in 2002 with the ^11^C-labeled Pittsburgh compound B ([^11^C]PIB, Figure 1), which is derived from the Aβ staining agent thioflavin-T (Klunk et al., 2004). [^11^C]PIB has a relative high selectivity for Aβ of all forms from soluble oligomers to insoluble fibrils and plaques over other pathologic proteins such as tau and α-synuclein. Both visual inspection and quantitative analysis demonstrated higher cortical retention in AD patients than in cognitively intact subjects, especially in the orbitofrontal cortex, inferior parietal cortex, posterior cingulate cortex and precuneus, which resembled the pattern found in immunohistochemical studies (Rowe and Villemagne, 2011). To date, [^11^C]PIB is still the best and most widely used Aβ PET tracer and regarded as the gold standard.

The short radioactive half-life of ~20 min for the carbon-11 nuclide limits the use of [^11^C]PIB to institutions with on-site cyclotrons, thus inspiring the development of ^18^F-labeled Aβ radiotracers. Three of such tracers with favorable binding and imaging properties, [^18^F]florbetapir ([^18^F]AV-45), [^18^F]florbetaben ([^18^F]AV-1, [^18^F]BAY-94-9172), and [^18^F]flutemetamol ([^18^F]GE-067) (Figure 1), have since been approved by the United States Food and Drug Administration (FDA) for clinical diagnosis and differential diagnosis of AD (Barthel et al., 2011; Lister-James et al., 2011; Curtis et al., 2015). [^18^F]NAV4694 ([^18^F]AZD4694), another promising ^18^F-labeled tracer with rapid pharmacokinetics and lower non-specific binding in the cerebral white matter, is now in clinical trials (Cselenyi et al., 2012; Therriault et al., 2021). These tracers, with longer radioactive half-life of ~110 min, are suitable for long-distance distribution and thus can be more widely used in the clinics. They are presumed to yield less noisier images and therefore more precise quantitation of minute cerebral Aβ accumulation in the early stage of the disease, due to more abundant radioactivity counts in the later period of scan. Notably, imaging protocols provided for each tracer are quite different from one another in terms of scanning window as well as visual interpretation and quantitative analysis of the images (Mallik et al., 2017).

### Imaging Research Findings and Clinical Relevance

In general, cortical Aβ retention detected by PET imaging is in good correlation with immunohistochemical staining of amyloid plagues at autopsy or biopsy brain samples (Clark et al., 2012; Rinne et al., 2012; Curtis et al., 2015; Sabri et al., 2015a). This qualifies the application of Aβ PET imaging as a non-invasive tool for *in vivo* detection of cortical Aβ deposition in the living brain. In the clinical daily-routine context, Aβ PET imaging provides plenty valuable information for the purpose of differential diagnosis between AD dementia and dementia disorders associated with non-Aβ pathologies such as frontotemporal lobe dementia (FTLD), which is sometimes indistinguishable from AD by neuropsychological assessments and conventional structural imaging modalities only (Rowe et al., 2008).

On the other hand, it has been emphasized that an amyloid-positive PET merely reflects the existence of amyloid neuropathology *in vivo* and does not necessarily guarantee a diagnosis of AD, concerning the fact that many otherwise cognitively normal subjects (even young healthy volunteers) have been found to be amyloid-positive judging from PET results, and that cognitive decline is more likely due to factors other than amyloid pathology in dementia with Lewy bodies (DLB) or in some co-morbid situations (Johnson et al., 2007; Ossenkoppele et al., 2015; Petrou et al., 2015). It has also been debated whether a negative amyloid scan could exclude the possibility of AD (Jack et al., 2016), since evidence of neurodegeneration in the absence of amyloid pathology challenges the proposed AD progression scheme (Sperling et al., 2011).

The ability of PET imaging with Aβ tracers to predict conversion from MCI due to AD, the prodromal stage of the disease defined by different diagnostic guidelines (McKhann et al., 2011; Dubois et al., 2014), to AD dementia has also been extensively investigated. Roughly 70% of amyloid-positive MCI patients converts to AD dementia within 3 years (Okello et al., 2009a). As compared to [^18^F]FDG PET, amyloid PET has higher sensitivity but relatively lower specificity (Teipel et al., 2015), which is consistent with the finding that amyloid accumulation commences at least a decade before the worsening of synaptic activity and brain function to a clinically significant level where amyloid deposition reaches a plateau (Jack and Holtzman, 2013; Jack et al., 2013a). Correlation of Aβ retention to cognitive performance has been shown to be greater in MCI and cognitively intact subjects than in AD dementia patients (Pike et al., 2007, 2011; Villemagne et al., 2008), which could also be explained by the hypothesized plateau model. Therefore, it is not surprising that regional hypometabolism and amyloid deposition in the temporoparietal regions are closely associated with each other whereas those in the frontal lobe, a region affected only in advanced AD, are not (Edison et al., 2007; Cohen et al., 2009).

The significance of Aβ PET in subjects without objective evidence of cognitive decline is emerging most recently. Large-scale comprehensive studies have shown that Aβ positivity is linked to high progression risk in subjective cognitive decline (SCD) and is associated with age and family history other than sex, education, marital or retirement status and self-reported lifestyle factors (Papp et al., 2020; Sperling et al., 2020). Longitudinal follow-up studies suggest that the duration of Aβ existence might be more important than age or binary result in affecting both deteriorating rate and the final status of cognition through elevated entorhinal tau burden (Hanseeuw et al., 2019; Koscik et al., 2020). Even if Aβ burden detected by PET is below threshold, its value is still positively correlated to the subject's future risk of cognitive decline (Guo et al., 2020).

### The Centiloid Scaling Project

As the utilization of amyloid PET in clinical trials and research expands and multiple tracers are available for such imaging applications, the urgent need for inter-tracer standardization and for multi-center collaboration and longitudinal comparison drove the launch of the centiloid scaling project (Klunk et al., 2015). According to the concept of the project, one institute can follow a multi-step regime to create a scaling from 0 (young healthy controls) to 100 (typical AD patients) using its own amyloid PET data (Rowe et al., 2016). In this way a universal cutoff value could then be directly or indirectly applied in multi-center imaging and/or longitudinal studies to allow for inter-site/inter-tracer comparisons. The study group of the centiloid project has now made progress in the derivation and verification of converting formula, enabling the translation of non-[^11^C]PIB Aβ PET semi-quantitative values to standardized [^11^C]PIB counterparts (Rowe et al., 2017; Battle et al., 2018; Bourgeat et al., 2018; Navitsky et al., 2018). The authenticity of the centiloid approach has been confirmed neuropathologically (Amadoru et al., 2020).

### Discussion

The amyloid cascade theory has been the predominant theory of AD etiology and drove the development of anti-Aβ therapeutics in the last 3 decades. The availability of Aβ PET imaging tracers and its applications in AD imaging have indicated that amyloid pathology may be a high risk factor for future cognitive decline. However, increasing evidence also indicates that cortical amyloid is not specific for the presence of cognitive symptoms, thus affecting the positive predictive value of Aβ PET imaging. Among populations without dementia, the prevalence of cerebral amyloid pathology as determined by Aβ PET imaging or cerebral spinal fluid (CSF) Aβ measurement is associated with age (Jansen et al., 2015), e.g., 33% of healthy elderly individuals have significant levels of Aβ deposition without apparent clinical symptoms (Rowe et al., 2010). Therefore, Aβ deposition alone cannot explain AD pathogenesis and progression. Repeated failures of clinical trials for many anti-Aβ drug candidates have dampened the hope for their efficacy as disease-modifying therapeutics. Nonetheless, it should be kept in mind that Aβ PET imaging will still remain the gold standard to investigate disease mechanisms as it provides information regarding the topography of Aβ lesions. Although there have been no successful anti-Aβ drugs up to date, Aβ PET imaging has provided useful outcome measures for anti-Aβ therapeutics in clinical trials (Salloway et al., 2014; Honig et al., 2018; Wessels et al., 2020).

## PET Tracers for Imaging TAU Tangles

### Overview

In addition to the β-amyloid peptides, microtubule-associated protein tau (MAPT), or simply tau protein, together with its misfolded products, has been more thoroughly studied in recent years to explore its relationship with AD. Similar to the case of senile plaques formed by Aβ, the formation of neurofibrillary tangles (NFTs) by paired helical filaments (PHFs) is also a neuropathological hallmark of AD (Braak and Braak, 1997). PHFs are aggregated by misfolded hyperphosphorylated tau protein whose binding affinity with the microtubules is weakened, causing neuronal cytoarchitecture breakdown and dysfunction (Hoover et al., 2010; Spillantini and Goedert, 2013).

With the repeated failures of anti-Aβ therapeutics in large scale clinical trials, the focus was shifted from Aβ to tau on the development of AD therapeutics and imaging agents (Giacobini and Gold, 2013). However imaging tau *in vivo* is more challenging than imaging Aβ. Tau protein has six unique isoforms characterized by the number of repeats of its microtubular binding domains, and multiple secondary/tertiary structures differentiated by the shape of the filaments (Spillantini and Goedert, 2013). In addition to its much lower abundance compared to Aβ peptides in the brain, MAPT's intraneuronal property demands the qualified tracer to cross neuron cell membrane in addition to the blood brain barrier. These factors collectively hamper the screening and identification of sensitive and specific compounds. Nevertheless, substantial progress has been made in overcoming these inherent obstacles, and preliminary studies have shown encouraging results worthy of the efforts (Hall et al., 2017; Leuzy et al., 2019).

### Tracer Development

PET imaging of fibrillary tau traced back to about the same time for amyloid, with [^18^F]FDDNP arguably as the earliest tracer (Agdeppa et al., 2001). Indeed, this tracer labels both amyloid plaques and fibrillary tau tangles *in vivo*, but this property is also its biggest disadvantage, as it has comparable affinity for both amyloid and tau proteins, i.e., a lack of selectivity for either target. The first selective tau tracer [^18^F]THK523 was developed by Tohoku University of Japan in 2005 (Okamura et al., 2005). Later structural modifications led to the development of other tracers in the THK family: [^18^F]THK5105, [^18^F]THK5117, [^18^F]THK5317, and [^18^F]THK5351, with improved binding and *in vivo* pharmacokinetic properties (Okamura et al., 2013; Chiotis et al., 2016; Betthauser et al., 2017). However, tracers in this family were later found to have notable off-target binding to monoamine oxidase-B (MAO-B), which greatly limited their utility in imaging of tauopathies including AD (Ng et al., 2017).

**Figure 2:**
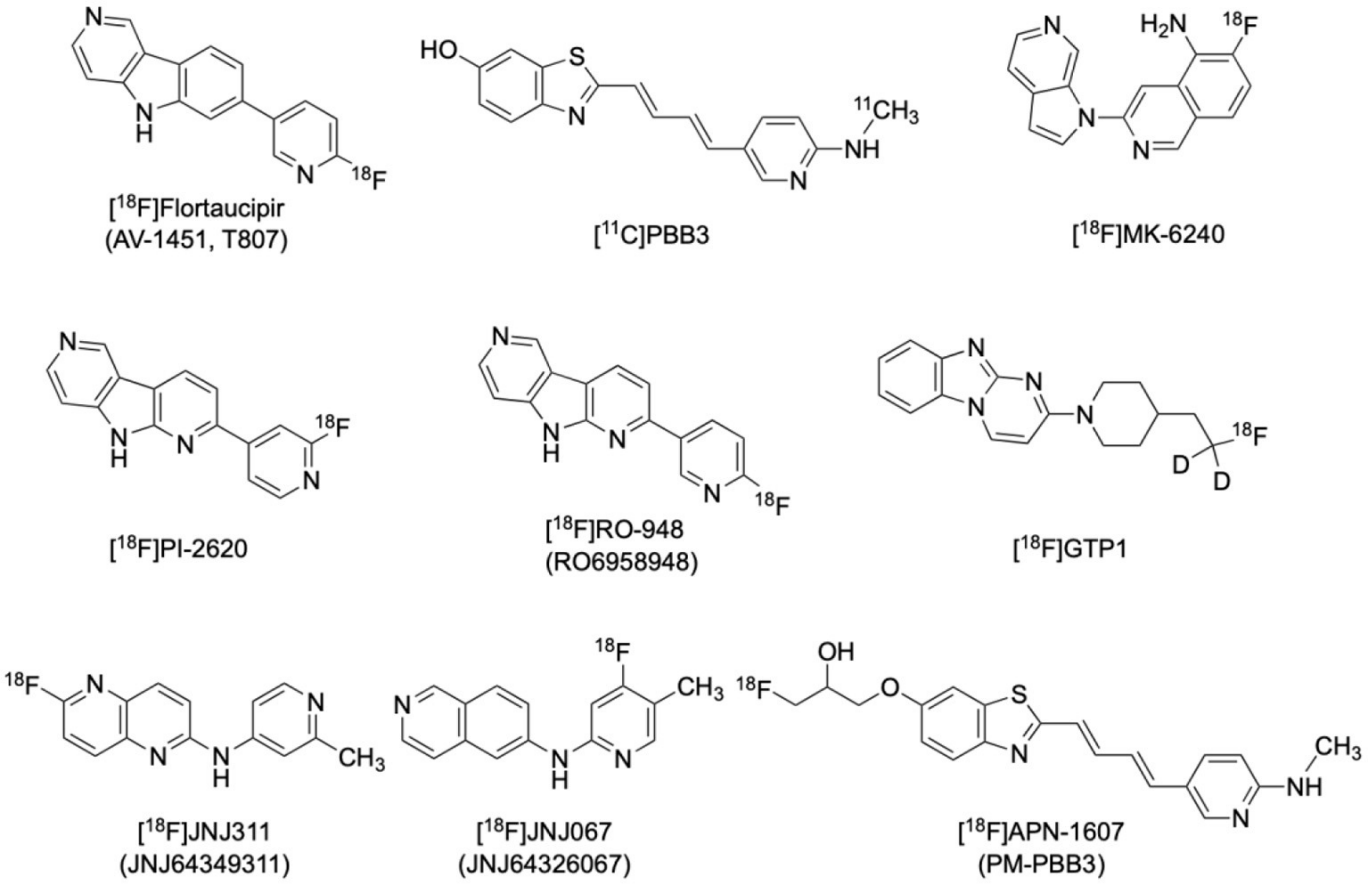
Structures of representative PET radiotracers for tau imaging.

[^18^F]Flortaucipir ([^18^F]AV1451, or formerly [^18^F]T807) is currently the most applied and the only FDA-approved tau radiotracer (Figure 2) (https://www.fda.gov/drugs/drug-approvals-and-databases/drug-trial-snapshot-tauvid). It has a 25-fold higher affinity for tau than Aβ, as well as favorable kinetics for both uptake and washout in the brain without radioactive metabolites penetrating the blood-brain-barrier (Xia et al., 2013). [^18^F]flortaucipir has higher affinity to PHFs over straight filaments (SF), and to combined 3-repeat (3R) and 4-repeat (4R) isoforms over 3R or 4R isoforms alone, making it more suitable for imaging AD pathology than non-AD tauopathies such as progressive supranuclear palsy (PSP) and corticobasal degeneration (CBD) (Lowe et al., 2016). It is noted that suspected minor off-target binding to MAO-A in the basal ganglia and substantia nigra would limit the application of [^18^F]flortaucipir in imaging Parkinsonian tauopathies (Ono et al., 2017).

[^11^C]PBB3 is another selective tau tracer that has also been thoroughly studied in various tauopathies including AD (Figure 2). [^11^C]PBB3 binds to both 3R and 4R tau isoforms and its affinity for tau is over 40 times higher than that for amyloid, making it suitable for imaging of various tauopathies *in vivo* (Kimura et al., 2015). Minor structural modifications then afforded [^18^F]APN-1607 (PM-PBB3) and [^18^F]AM-PBB3, the next generation members of the PBB3 family. [^18^F]APN-1607, (Figure 2) has recently been reported to possess more favorable pharmacokinetics and provide higher gray-matter/white-matter contrast (Hsu et al., 2020; Su et al., 2020; Tagai et al., 2021).

The clinical development of several newer, second generation selective tau tracers are ongoing, including [^18^F]PI2620, [^18^F]MK6240, [^18^F]GTP1, [^18^F]RO-948 (RO6958948), [^18^F]JNJ-311 (JNJ64349311), and [^18^F]JNJ-067 (JNJ-64326067) (Figure 2). Designers of these new generation tracers are focusing on improving *in vivo* characteristics such as higher selectivity, faster brain penetration/washout, and less off-target binding. Preliminary studies for these newer tracers have shown promising results (Declercq et al., 2017; Kuwabara et al., 2018; Guehl et al., 2019; Rombouts et al., 2019; Teng et al., 2019; Schmidt et al., 2020).

Off-target binding has been a universal concern for tau tracers since the early [^18^F]FDDNP was found to be non-selective to both amyloid and tau (Thompson et al., 2009). In PET imaging of neurodegeneration, selectivity of tau tracers over other pathological proteins such as Aβ, α-synuclein, and TDP-43 would always require validation. Similarity of the secondary/tertiary structures of the binding sites of these proteins makes it difficult to find a truly selective and specific probe, not to mention the complexity introduced by potential comorbidity of the neurodegenerative disorders. In addition, off-target binding in the central nervous system (CNS) has been widely examined across the tau tracers. It is now known that MAO-A and MAO-B are the most frequent off-targets whose secreting neurons are highly overlapped with Parkinsonism-related brain regions (Lowe et al., 2016; Bischof et al., 2017; Okamura et al., 2018). Choroid plexus is frequently found to be a tissue with apparent off-target binding, the mechanism of which is yet unclear (Ikonomovic et al., 2016). It is postulated that melanin, neuromelanin, mineralized structures and hemorrhagic lesions can also cause off-target binding of tau tracers in various locations. There is also debate that the suspected “off-target” binding may be reflecting true tau-binding, or binding to some specific targets yet to be identified (Ikonomovic et al., 2016; Passamonti et al., 2017). These characteristics pose substantial difficulties to clinical differentiation and potential post-treatment evaluation (Passamonti et al., 2017).

Recent cryo-electron microscopic structure discoveries of AD tau filaments may provide new insights for the binding interactions between various tau tracers and tangles, and spur refinements on the design of novel, subtype-selective tau tracers (Fitzpatrick et al., 2017).

### Imaging Research Findings and Clinical Relevance

According to the amyloid cascade hypothesis, neurodegeneration characterized by misfolded tau tangle aggregation is the downstream event secondary to major amyloid deposition (Jack et al., 2013b). In contrast to the globally elevated pattern seen in brain amyloid PET of AD dementia patients, the spatiotemporal distribution of tau tracers follows a typical neuropathological sequence of spreading (Braak's stage), as have been demonstrated in both cross-sectional and longitudinal studies in a wide-range of subjects from advanced AD dementia patients to cognitively intact elderly controls (Rabinovici and Jagust, 2009; Johnson et al., 2016; Wooten et al., 2017; Villemagne et al., 2018). This quantitative and sequential connection indicates that this *in vivo* tangle spreading trajectory may reflect not merely neuronal dysfunction but also disease progression. In parallel to postmortem immunohistochemical findings, *in vivo* tau deposition varies amongst the brain regions, starting from the medial temporal lobe, i.e., hippocampus and entorhinal cortex (Braak stage I–II), to the adjacent neocortices (Braak stage III–IV), and finally to the entire brain (Braak stage V–VI) (Cho et al., 2019; Leuzy et al., 2019; Baek et al., 2020; Fleisher et al., 2020). As expected, clinical manifestation of AD is closely related to tau retention in the responsible brain regions. For instance, while temporal lobe deposition correlates well with memory performances, frontal lobe retention strongly correlates with execution and global cognition (Ossenkoppele et al., 2016; Bejanin et al., 2017; Shimada et al., 2017).

According to current experience with all the available tau tracers, AD is not likely to be the diagnosis of a tau-negative individual, while non-tauopathies can be basically excluded from the cause of cognitive decline for a tau-positive individual (Bischof et al., 2017). Non-AD tauopathies such as FTLD, PSP and CBD, however, manifest different distribution patterns and trajectory from AD involving different sub-regions in the brain stem and basal ganglia as well as cortical regions, which could be of additional differential diagnostic value in the clinical context (Bischof et al., 2017; Leuzy et al., 2019). Recently, tau deposition in cognitively intact healthy elderly subjects has also been revealed by PET and confirmed by autopsy results. This phenomenon is coined primary age-related tauopathy (PART) and believed to produce only minute, if any, clinical symptoms (Crary et al., 2014). In the absence of amyloid, PART alone is believed to be insufficient to develop memory decline (Harrison et al., 2019). Its relationship to suspected non-amyloid pathology (SNAP) has also been proposed, which presumably underlies the preclinical abnormalities during the development of AD (Jack, 2014).

**Figure 3:**
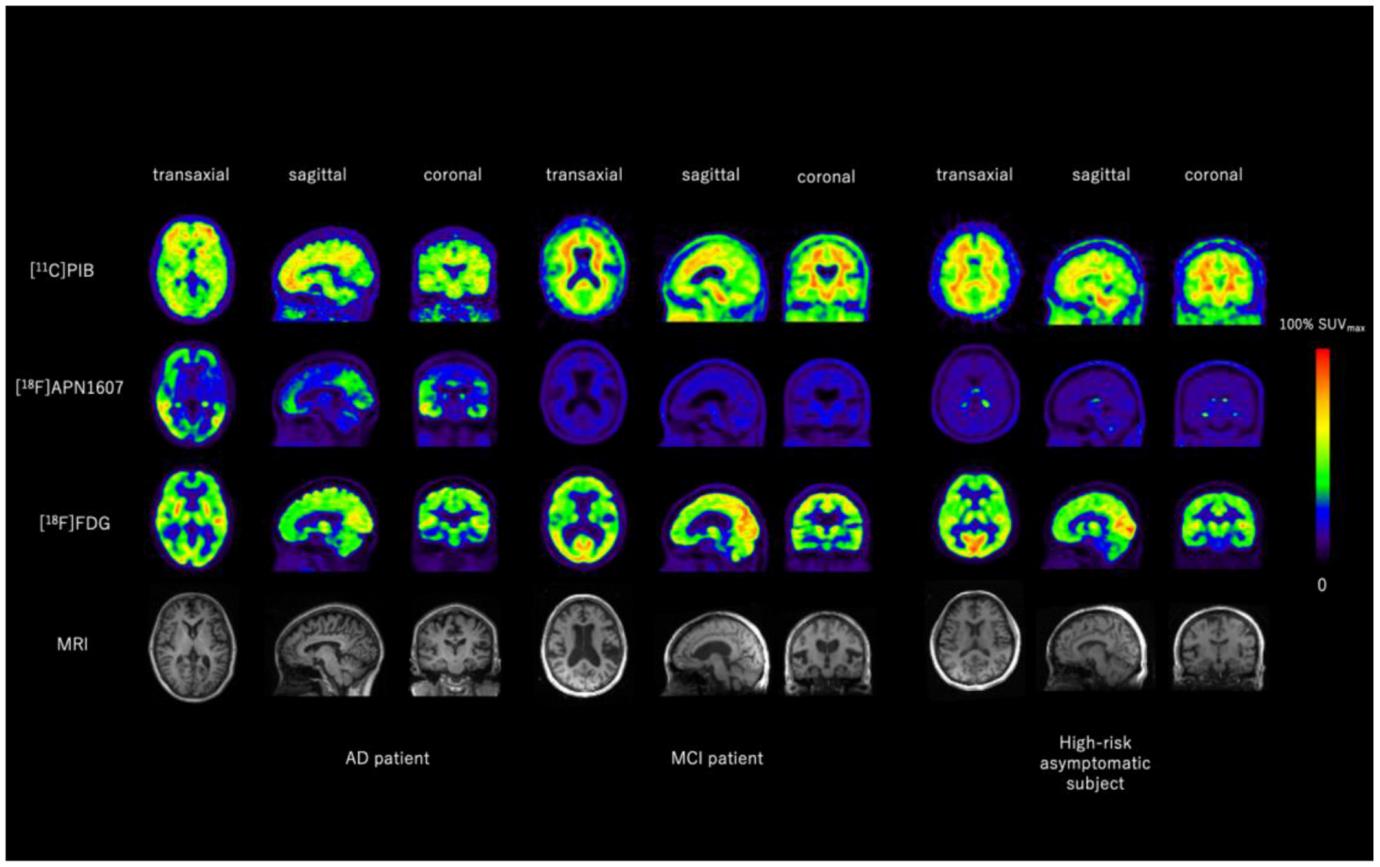
Representative images of [^11^C]PIB, [^18^F]APN1607, and [^18^F]FDG PET and MRI of an AD patient, an MCI patient and a high-risk asymptomatic subject. Aβ was positive for the AD patient and equivocal for the MCI patient and the high-risk asymptomatic subject. Typical Braak Stage V–VI tau deposition was seen in the AD brain, whereas no tau deposition was seen in the brain of the MCI patient or the high-risk asymptomatic subject. Typical AD-like glucose-hypometabolism was seen in AD brain including posterior cingulate cortex, parietal, temporal, and pre-frontal cortices, whereas most of these regions are relatively spared in the brain of MCI patient and high-risk asymptomatic subject. Images provided by PET Center, Huashan Hospital, Fudan University.

Hypometabolism in brain regions that are commonly affected in advanced AD, including posterior cingulate cortex, precuneus and temporoparietal association cortex, represents synaptic dysfunction of the neurons in the course of AD (see below section on FDG imaging of glucose metabolism). Cortical tau tracer deposition can be seen in the same areas, establishing a typical topographic “yin-yang” offset between the two biomarkers. Further, correlation between these two imaging findings grows stronger as tau burden increases (Figure 3) (Whitwell et al., 2018; Lu et al., 2020). These unique phenomena demonstrate outstanding pathophysiological coherency of excessive MAPT retention and subsequent destruction of cytoarchitecture, resulting in synaptic metabolic deficit. On the other hand, multi-modality studies also reveal that the correspondence between frontal hypometabolism and medial temporal tau retention is present independent of amyloid deposition, which can be interpreted as aging-related.

The relationship between tau aggregation and cortical atrophy as demonstrated by structural MRI is similar to that between tau aggregation and hypometabolism. However the former was found to be weaker than the later, especially in conditions other than mild AD (Sepulcre et al., 2016; Iaccarino et al., 2018). In addition to local correlation, tau deposition in the parietal lobe and precuneus can also be correlated to medial temporal lobe atrophy (Shimada et al., 2017). These observations seem plausible due to the fact that substantial structural alterations often relate to the later stage of the disease.

In general, CSF phosphorylated tau (p-tau, representing misfolded tangle formation) and total tau (t-tau, representing neurodegeneration) levels parallel *in vivo* tau tracer binding (Mattsson et al., 2018; Leuzy et al., 2019; Okafor et al., 2020). However, discordant results have also been found in small subgroups, possibly due to varying detectability around the CSF threshold and uneven rate of tau tangle formation in different disease stages (Murray et al., 2015; Thal et al., 2015). Although it is believed that elevated CSF tau levels precede tau imaging manifestation, tau PET has the advantage of visualization and topographic quantitation capacity, as well as relative non-invasiveness (Wolters et al., 2020).

### Discussion

The research and development of tau PET tracers has gained undeniable progress in the last few years. Although some issues remain, especially isoform selectivity and other potential off-target binding, the applications of these tracers in AD imaging have provided, and are expected to continue to provide valuable information on the time course and topography of tau tangles in AD, and the correlation with cognitive dysfunction. Longitudinal and cross-sectional multi-target and multi-modality studies are encouraged to further elucidate the role of tau in the course of AD, as well as its interaction and relationship with Aβ deposition, synaptic dysfunction, brain atrophy, and other pathological biomarkers. Another important application of tau imaging is its utility for patient selection and endpoint measurements in phase 2 and phase 3 clinical trials of disease-modifying anti-tau treatments that have been gaining increasing impetus (Giacobini and Gold, 2013).

Coming from different compound families, the currently available tau tracers have different affinities for the various MAPT isoforms or tangle structures, hence distinctive topographic binding patterns in the same tauopathy. Selectivity to isoforms and structures could be a future direction to design new probes as 3R-specific or 4R-specific. We could therefore foresee a future tau imaging landscape where different tracers “rule” their own pieces of territory (i.e., specific tauopathy characterized by specific isoform or structure) in case that the correlation of tau imaging results similar to the “Aβ centiloid” is not achievable.

## PET Tracer for Imaging Glucose Metabolism: [^18^F]FDG

### Overview

As a radionuclide-labeled analog of glucose, the major metabolic substrate of neurons, [^18^F]fluorodeoxyglucose ([^18^F]FDG, or FDG) has long been used in the investigation of CNS disorders to reflect neuronal degeneration and injury. In fact the first report of this most widely used tracer was a brain imaging study (Phelps et al., 1979), although now the majority of its clinical application is for oncological purposes. The characteristic AD pathology (McGeer et al., 1986) and neurodegenerative changes (Mielke et al., 1996; Scholl et al., 2011) are associated with cortical hypometabolism demonstrated in pre-mortem FDG PET imaging. Clinical application of FDG PET in AD lies mainly in differential diagnosis from other causes of dementia, as well as treatment effect evaluation of disease-modifying or progression-slowing therapies. Clinical value of FDG PET has also been investigated for its predictive ability for conversion to AD dementia of high-risk subjects assumed to be in the prodromal or asymptomatic stage.

### AD Dementia Patients

The characteristic manifestation of FDG PET in AD dementia is hypometabolism in the posterior part of cerebrum including the posterior cingulate cortex (PCC), precuneus (PrC), and parietotemporal association cortices such as the angular gyrus. These regions are the most discriminating components of AD-specific cerebral hypometabolic pattern as well as strong indicators of disease severity and progression. It is of significant differential value that PCC and PrC are substantially spared in other minor causes of senile dementia including frontotemporal lobe dementia (FTLD), dementia with Lewy bodies (DLB), Parkinson's Disease dementia (PDD), and vascular dementia (VaD). The hippocampus and entorhinal cortex are involved in the earliest stages of AD according to neuropathological findings based on NFT formation (Braak and Braak, 1997), however glucose metabolism in these areas cannot be readily distinguished between AD patients and normal controls. This is largely due to the frequent presence of medial temporal hypometabolism even in normal aging, and the partial volume effect (PVE) correlated with regional cortical atrophy. Frontal lobe hypometabolism is often associated with advancement, executive dysfunction, or atypical behavior in AD cases, although it could also be observed in normal aging. Occipital lobe hypometabolism is related to the posterior cortical atrophy (PCA) subtype of AD, but care should be taken when differentiating dementia types because this is also a prominent sign of DLB.

### MCI Patients

Early differentiation of MCI due to AD is crucial since early therapeutic intervention is indicated to be beneficial at least in slowing disease progression (Vellas et al., 2007; Molinuevo et al., 2011). It should be noted, though, that the etiology of MCI is heterogeneous, meaning that approximately half of the patients convert to dementia other than AD, or do not convert at all (Rowe et al., 2010; Bennett et al., 2012; Frisoni et al., 2017). Therefore, whether FDG PET has discriminative and predictive ability for MCI patients is of great clinical relevance.

Hypometabolism revealed by FDG PET in typical AD-affected brain regions including the inferior parietal lobe, precuneus and posterior cingulate cortex is present early in prodromal AD, namely MCI stage. In a meta-analysis comparing the accuracy of 3 different imaging modalities, FDG PET (sensitivity = 88.8%, specificity = 84.9%) exhibits higher sensitivity and higher specificity than cerebral blood flow SPECT (sensitivity = 83.8%, specificity = 70.4%) and structural MRI (sensitivity = 72.8%, specificity = 81%) in terms of predicting short-term conversion to AD dementia (Yuan et al., 2009). Interestingly, FDG PET performs better in excluding non-converters than [^11^C]PIB Aβ PET (specificity: 74.0 vs. 56.2%), while its sensitivity is lower (sensitivity: 78.7 vs. 93.5%) (Zhang et al., 2012). This could be explained by the compensatory mechanism of preserved cerebral synaptic function against amyloid burden. Aside from the advantage of accurate short-term predictive ability, FDG PET can also exclude other potential etiology underlying MCI such as FTLD and DLB, in contrast to other biomarkers including CSF Aβ_1−42_ and t-tau/p-tau assays (Arbizu et al., 2018).

The value of FDG PET in MCI has been acknowledged in existing diagnostic criteria (Albert et al., 2011; Dubois et al., 2014). Meanwhile, future researches in this field are encouraged to overcome the various current limitations in terms of methodology normalization, gold standard verification, and effectiveness/economy evaluation (Arbizu et al., 2018).

### High-Risk Asymptomatic Subjects

People with subjective cognitive decline (SCD) rather than objective evidence of cognitive impairment, subjects burdening amyloidosis, and family members of AD patients expressing PSEN1/2 or APP mutation were reported to have higher tendencies to develop to AD dementia (Bateman et al., 2011; Villemagne et al., 2011; Wolfsgruber et al., 2016). It is hypothesized that neurodegenerative alterations could have be silently undergoing in these subjects.

Various confounding factors lead to heterogeneity of the underlying etiology for SCD, leaving controversy in the regional metabolic manifestation of typical AD-affected areas in this subgroup (Scheef et al., 2012; Brugnolo et al., 2014; Van Der Gucht et al., 2015). Although amyloid-positive asymptomatic subjects, as defined by CSF Aβ_1−42_ or amyloid PET, will have higher risk of converting to AD in a life-long period, the utility of FDG PET reflecting neurodegeneration in this population is still not comparable to that in MCI patients, due to its poor performance in prediction of short-term conversion (Villemagne et al., 2011). Glucose metabolic abnormalities prior to the onset of clinical symptoms were observed in asymptomatic carriers of mutated APP and PSEN1/2 genes (Mosconi et al., 2008; Benzinger et al., 2013), who theoretically would suffer from dementia eventually. Nonetheless, the relationship between cerebral hypometabolism and time-to-conversion cannot be easily concluded in the absence of well-designed longitudinal studies.

In view of the above-mentioned facts, it is not recommended to clinically apply FDG PET to asymptomatic subjects with only one risk factor for diagnostic or prognostic purposes. Further investigation is needed to verify whether FDG PET is useful for individuals with multiple risk factors (Mosconi et al., 2008; Vannini et al., 2017).

### Treatment Monitoring

FDG PET has been used as an imaging biomarker for outcome assessment in multiple clinical trials of AD therapeutics (Hoyer, 2002; Landau et al., 2011; Herholz, 2012). Global as well as sub-global or regional FDG uptake alterations following medication or surgical treatment has been observed in multiple clinical research studies (Nordberg et al., 1992; Heiss et al., 1994; Mega et al., 2001; Potkin et al., 2001; Tune et al., 2003; Schmidt et al., 2008; Tzimopoulou et al., 2010; Craft et al., 2012; Smith et al., 2012).

### Discussion

FDG PET has been most widely used in dementia research and as an important adjunct imaging tool in the diagnosis of AD. On the whole, FDG PET enables early diagnosis of AD and thus early therapeutic intervention, as well as treatment strategy optimization in a large proportion of cases (Laforce et al., 2010; Elias et al., 2014). Delaying of disease progression and prevention of life quality deterioration as instructed by FDG PET can help lessen the overall healthcare expenditure (Banerjee and Wittenberg, 2009; Getsios et al., 2012).

Although quite a few critical issues in the utility of FDG PET for AD diagnosis have already been resolved, there are still many others that require verification with larger cohorts and better-designed trials (Garibotto et al., 2017).

## PET Tracers for Imaging Neuroinflammation

### Overview

Increasing evidence has helped with the formation of neuroinflammation hypothesis of AD etiology (McGeer and McGeer, 2010; Morales et al., 2014). Similar to the cases in other systems of the human body, it is now believed that the impact on CNS under different phases of the inflammatory process could be different. While acute inflammation could be protective to the brain against the harmful effects of invading pathogens and traumatic injuries, consistent stimulation by inflammatory factors could, on the other hand, be detrimental to the neurons and eventually induce neurodegeneration and loss of cognitive functions (Wyss-Coray and Mucke, 2002; Mrak and Griffin, 2005). It is also suggested that the neuroinflammation underlying AD starts from the earliest stages without any obvious clinical symptoms, and lasts to the end stage of the disease (Vehmas et al., 2003; Hoozemans et al., 2005). Therefore it is hypothesized that the clinical onset of AD dementia could be partly prevented or postponed by anti-inflammation interventions, which is based on results from retrospective observation studies and prospective trials (McGeer et al., 1996; Hoozemans et al., 2011). Multiple pathophysiological factors can trigger the process of immunoactivation in the CNS, in which complements, cytokines, growth factors, reactive oxygen species, microglia and astrocytes participate (Barger and Harmon, 1997; Akiyama et al., 2000). PET radiotracers have been developed to image these neuroinflammatory targets *in vivo* (Zimmer et al., 2014; Varley et al., 2015).

### Tracer Development

The 18-kDa translocator protein (TSPO) is a mitochondrial protein. Under normal circumstances, it is low-expressed in specific brain regions in the microglia, the monocyte-macrophage-dendritic cell family member majorly active in the CNS and comprising ~15% of the non-neuronal cells within. TSPO is found to participate in amino acid and cholesterol transportation, and to serve as a “switch” in activating microglia from the resting state in response to various stimuli including infection and traumatic injury (Streit et al., 2004). Pro-inflammatory cytokines and neurotoxic substances are released by significantly proliferated microglia after the induction of soluble or fibrillary Aβ and subsequent TSPO regulation, which could be related to the fact that amyloid plaques are found to be colocalized with activated microglia *in vivo* in the brain where massive neuronal destruction and brain atrophy occur (McGeer et al., 1988; Venneti et al., 2009; Schilling and Eder, 2011). These characteristics made TSPO the predominant target for imaging *in vivo* inflammatory process in AD.

**Figure 4:**
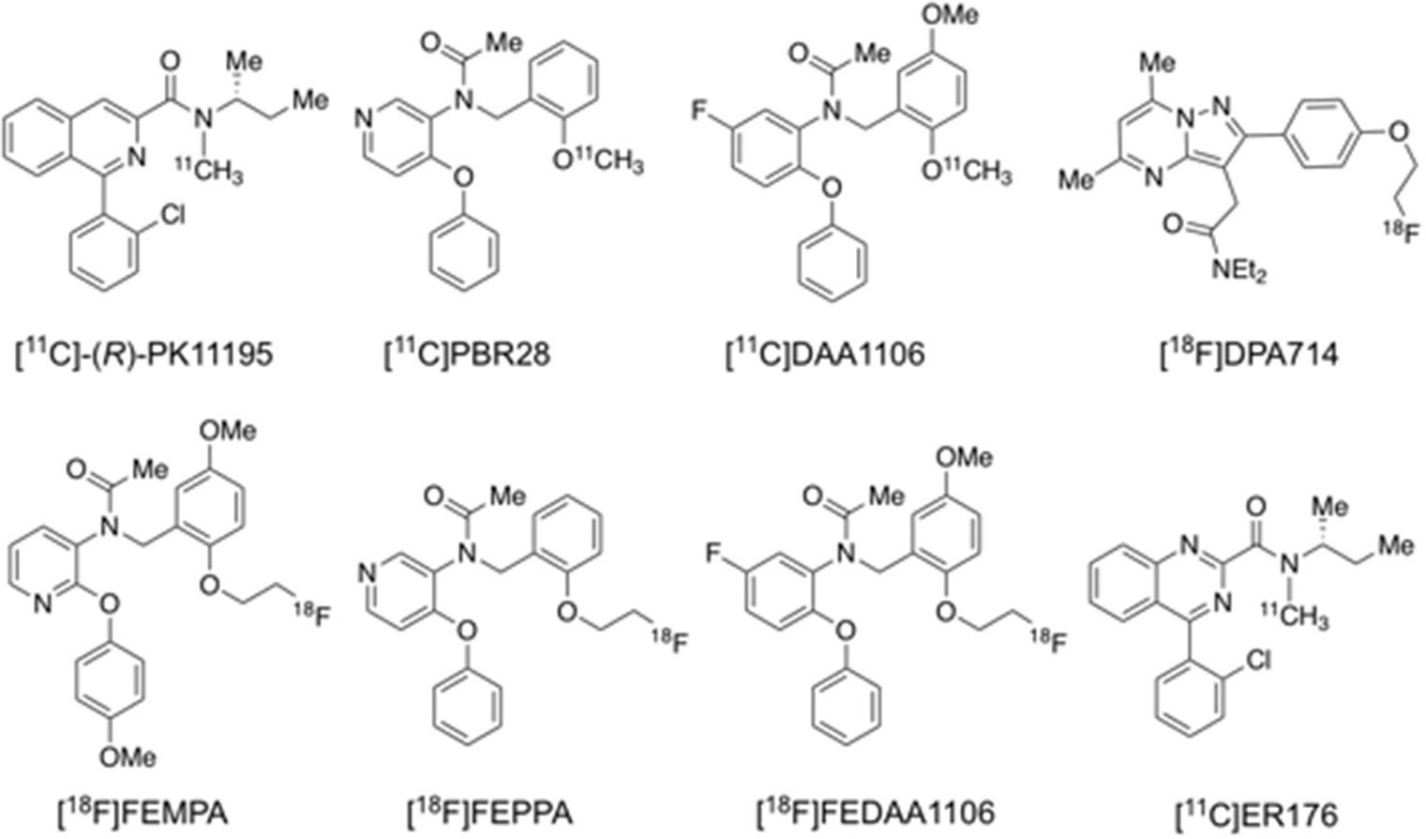
Structures of representative PET radiotracers for TSPO imaging.

[^11^C]PK11195 was the first successfully developed TSPO tracer for human PET imaging (Figure 4) (Cagnin et al., 2001), but it remains controversial whether its binding is correlated with amyloid deposition, probably due to its low specific binding signal and hence low sensitivity to detect small changes in TSPO under disease conditions (Edison et al., 2008; Wiley et al., 2009; Yokokura et al., 2011). To overcome the shortcomings of [^11^C]PK11195, a series of 2nd generation TSPO tracers were developed and evaluated, including [^11^C]PBR28, [^11^C]DAA1106, [^18^F]DPA713, [^18^F]DPA714, [^18^F]FEPPA, [^18^F]FEMPA, and [^18^F]FEDAA1106 (Figure 4; Varley et al., 2015; Calsolaro and Edison, 2016; Edison et al., 2018). Though the majority of these radioligands are more sensitive than [^11^C]PK11195, subsequent studies found that their brain uptake are regulated by the TSPO gene rs6971 polymorphism, thus requiring phenotyping of individual subjects to match imaging results to their TSPO affinity status (Owen et al., 2012). More recently, 3rd generation, putative “phenotype-insensitive” TSPO probes such as [^11^C]ER176 (Figure 4) are being developed and evaluated (Wadsworth et al., 2012; Ikawa et al., 2017).

Similar to the paradigm of TSPO imaging of microglial activation, MAO-B is found to be elevated in reactive astrocytes and chosen as the target for *in vivo* imaging of neuroinflammation. The selective MAO-B tracer [^11^C]deuterium-L-deprenyl ([^11^C]DED) has been applied to studies of neurodegenerative disorders including AD (Carter et al., 2012). Type-2 imidazoline receptor, a newly discovered target for imaging astrocyte, is now being evaluated for its potential in differentiating AD and control using [^11^C]BU99008 under ongoing trial (Wilson et al., 2019).

### Imaging Research Findings and Clinical Relevance

**Figure 5:**
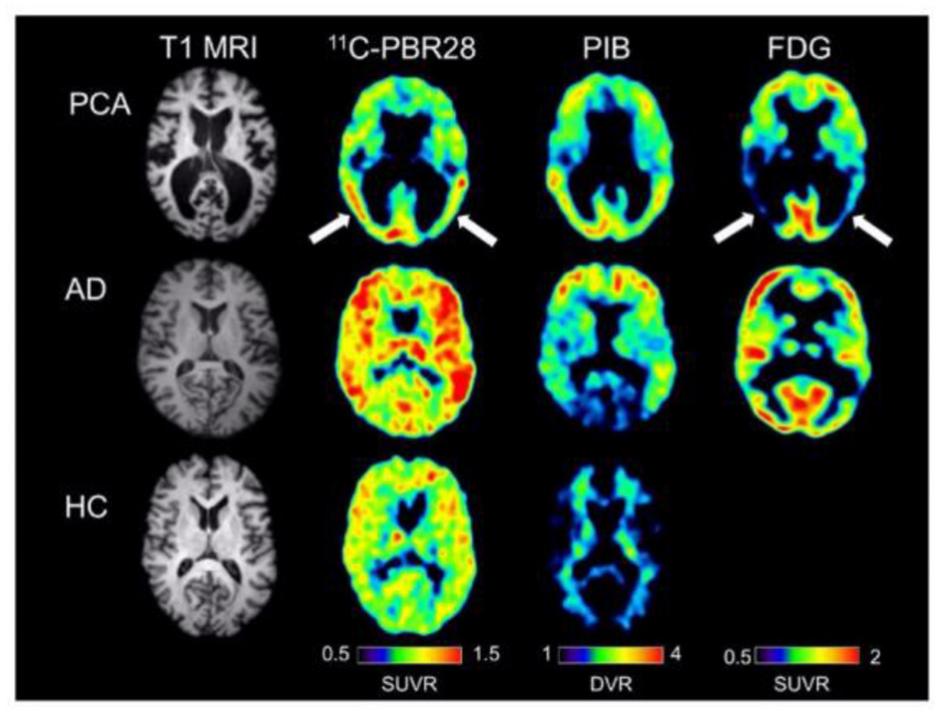
Single subject images from a patient with posterior cortical atrophy (top row), a patient with amnestic Alzheimer's disease (center row), and a healthy control subject (bottom row). The posterior cortical atrophy subject showed focal occipito-temporal [^11^C]PBR28 binding, with FDG hypometabolism in the same region (arrows). While the posterior cortical atrophy subjects showed occipito-temporal PIB binding, PIB binding was also found in frontal cortex. The subject with amnestic Alzheimer's disease showed more diffuse [^11^C]PBR28 binding, with occipital sparing on PIB and classic bilateral temporo-parietal hypometabolism on FDG imaging. The control subject showed low amounts of diffuse [^11^C]PBR28 binding and absence of cortical PIB [^11^C]PIB binding. Courtesy from Kreisl et al. (2016b).

*In vivo* TSPO imaging is generally able to discriminate between AD dementia patients and normal control subjects (Cagnin et al., 2001; Yasuno et al., 2008; Suridjan et al., 2015; Varrone et al., 2015; Hamelin et al., 2016; Kreisl et al., 2016a). The majority of the multi-modality studies report that regional TSPO binding correlates to [^11^C]PIB retention positively, and FDG uptake as well as cortical volume negatively (Edison et al., 2008; Yokokura et al., 2011; Kreisl et al., 2013, 2016b) (Figure 5). In most cases the extent of TSPO binding is associated with not only baseline cognitive performance, but also its deterioration over time (Edison et al., 2008; Okello et al., 2009b; Yokokura et al., 2011; Kreisl et al., 2013, 2016a). Elevated TSPO binding in advanced AD dementia patients is seen in various cortical regions following an anticipated distribution pattern (Cagnin et al., 2001; Hamelin et al., 2016). Apart from global discrimination, regional TSPO binding is correlated to attenuated corresponding brain function. One study uncovered an inverse correlation between visuospatial function and [^18^F]FEPPA binding in the parietal and posterior internal capsule, as well as correlation between language ability and binding in the latter region (Suridjan et al., 2015). Interestingly, early-onset AD (EOAD) is reported to be associated with higher TSPO binding than late-onset AD (LOAD), especially in the frontal and parietal cortices, suggesting greater microglial activation in the former condition (Kreisl et al., 2013). In addition, a longitudinal study revealed that TSPO binding in MCI converters to AD dementia is drastically different from that of non-converters (Kreisl et al., 2016a). These results are concordant with previous finding that chronic microglia participation may be associated to the brain in AD progression (McGeer and McGeer, 2010; Morales et al., 2014).

However, conflicting results are also present in various aspects. Absence of the expected correlation between TSPO binding and cortical amyloid retention or neuronal metabolism has been repeatedly reported (Okello et al., 2009b; Wiley et al., 2009; Schuitemaker et al., 2013). There are also studies showing the inability of TSPO tracers to discriminate healthy subjects from patients with MCI or even advanced dementia (Okello et al., 2009b; Wiley et al., 2009; Schuitemaker et al., 2013; Golla et al., 2015). Likewise, significant association between TSPO binding and memory performance or disease severity is not always found (Yasuno et al., 2008; Schuitemaker et al., 2013). Single-nuclide polymorphism of rs6971 has been discovered to regulate TSPO binding *in vivo* and may partly contribute to the conflicting findings so far, since binding adjusted to individual phenotyping shows increased discriminating accuracy (Suridjan et al., 2015). In addition to doubts about the sensitivity of current TSPO tracers to detect subtle alterations in the prodromal stages of AD, there are data relating higher binding of [^18^F]DPA714 to slower cognitive decline, suggesting a neuroprotective role of microglial activation perhaps in the early phase of the disease (Hamelin et al., 2016).

According to a collective study with both [^11^C]DED and [^11^C]PIB, astrocytosis is most profound in amyloid-positive MCI subjects, followed by advanced AD patients as well as amyloid-negative MCI subjects and healthy controls (Carter et al., 2012; Rodriguez-Vieitez et al., 2016). This PET finding suggests that astrocytic reaction diminishes after prodromal AD converts to dementia, and is supported by postmortem autoradiographic study showing highest binding of MAO-B radioligand in the earliest Braak stages (Gulyas et al., 2011).

### Discussion

It is universally acknowledged that neuroinflammation plays an active part in the course of AD. PET imaging of neuroinflammation targets have, in part, confirmed the correlation between Aβ deposition and elevated microglia/astrocyte activation/neuroinflammation. Nonetheless, in light of the complexity of both the process and components, questions such as “Is neuroinflammation beneficial at the beginning and harmful thereafter?” and “Is TSPO imaging and MAO-B imaging reflecting true activation of microglia and astrocytes?” remain to be answered. In order to obtain a clearer picture of the various aspects of inflammation in the CNS, further exploration is needed with some of the clues now at hand. First, microglia can be polarized to either M1, releasing neurotoxic substances, or to M2, releasing neuroprotective cytokines (Mosser and Edwards, 2008). Highly selective radioligands discriminating the two opposing activations will help answer these questions. Further, longitudinal studies, ideally tracking from the very early stage to the end stage of the disease across subjects, will be more informative than cross-sectional studies in mapping the time course and topography of microglia activation in disease progression. In addition, genotype-insensitive TSPO tracers under development require verification and validation in large-sample cohorts (Wadsworth et al., 2012; Zanotti-Fregonara et al., 2014; Ikawa et al., 2017). Finally, multi-modality imaging approaches will be helpful in revealing the interactions between neuroinflammation and various pathologic/pathophysiological components in AD.

## PET Tracers for Imaging Targets in the Cholinergic System

### Overview

The basal/rostral forebrain cholinergic pathways are believed to play an important role in a variety of neuropsychological functions including attention, consciousness and memory processing (Perry et al., 1999). Cholinergic replacement countering the loss of cholinergic neurotransmission in neurodegeneration is the theoretical basis of AD-treatment strategies using clinically approved medications (Bartus et al., 1982; Schliebs and Arendt, 2011). Cholinergic depletion and the resulting deficit in neuronal compensatory plasticity are confirmed in autopsy studies of AD patients (Bierer et al., 1995; Craig et al., 2011). PET imaging tracers targeting various aspects of cholinergic neurotransmission and metabolism can help us to better understand the role of cholinergic neuropathy and its interaction with other pathologic/pathophysiological components in the course of AD.

### Tracer Development

**Figure 6:**
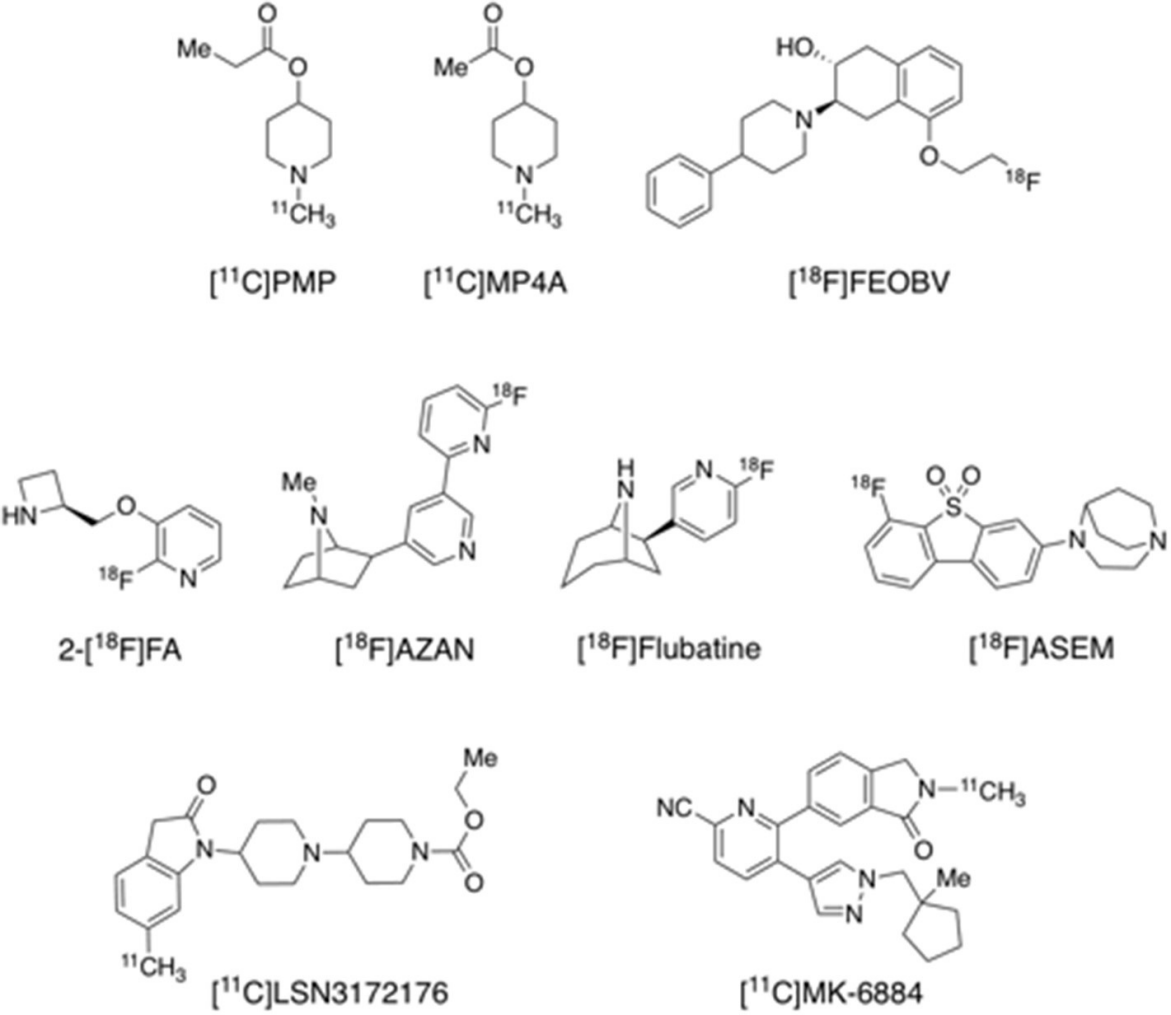
Structures of representative PET radiotracers for cholinergic targets.

Acetylcholinesterase (AChE) is the major target of AD medications, most of which are acetylcholinesterase inhibitors (AChEIs). These therapeutic agents, including galantamine, rivastigmine, tacrine and donepezil, block AChE to inhibit hydrolysis of ACh, thus increasing ACh level in the synaptic cleft. *N*-[^11^C]methyl-4-piperidinyl propionate ([^11^C]PMP) and *N*-[^11^C]methyl-4-piperidyl acetate ([^11^C]MP4A) are two selective substrates for AChE that have been successfully brought to *in vivo* human imaging research (Figure 6). Another presynaptic cholinergic PET tracer [^18^F]FEOBV, selective to the vesicle ACh transporter (VAChT), is reported to have been assessed in humans (Aghourian et al., 2017). Postsynaptic acetylcholine receptors can be classified into nicotinic acetylcholine receptors (nAChRs) and muscarinic acetylcholine receptors (mAChRs). While most of the previous human PET studies used α_4_β_2_ or non-selective nAChR tracers including [^11^C]nicotine, 2-[^18^F]F-A-85380 ([^18^F]2-FA), [^18^F]AZAN, and [^18^F]flubatine (also known as [^18^F]NCFHEB) (Figure 6) (Nordberg et al., 1990; Sabri et al., 2008, 2015b; Wong et al., 2013), successful development of selective tracers for new targets such α_7_ nAChR and M1 and M4 mAChR has been reported recently. Recent imaging evaluations in humans indicated that [^18^F]ASEM, [^11^C]LSN3172176, and [^11^C]MK-6884 (Figure 6) have appropriate kinetics and imaging properties and are promising tracers for their respective targets α_7_ nAChR and M1 and M4 mAChR (Hillmer et al., 2017; Wong et al., 2018; Masdeu et al., 2020; Naganawa et al., 2020a; Tong et al., 2020). [^18^F]ASEM and [^11^C]MK-6884 have been used in preliminary studies of α_7_ nAChR and M4 mAChR in AD (see below), while evaluation of M1 mAChR in AD is ongoing with [^11^C]LSN3172176 in our laboratories.

### Imaging Research Findings and Clinical Relevance

AChE imaging using [^11^C]PMP and [^11^C]MP4A showed reduced cortical binding in AD patients than in healthy controls, especially in regions innervated with cholinergic projections (Iyo et al., 1997; Kuhl et al., 1999). AChE binding is lower in AD patients than in healthy controls, in parallel with VAChT reduction, and is further decreased by treatment with AChE inhibitors donepezil, rivastigmine and galantamine (Kuhl et al., 1999, 2000; Shinotoh et al., 2001; Kaasinen et al., 2002; Kadir et al., 2008). However this induced inhibition is absent for nAChR binding, suggesting that allosteric modulation effect maintains ACh signaling by up-regulating nAChR but not AChE (Maelicke et al., 2001). The degree of treatment-induced decline in AChE binding is more prominent in the frontal cortex than in the temporoparietal cortices, and correlated to improvement in frontal lobe functions such as execution and attention rather than episodic memory. Correlation between AChE hydrolysis and hypometabolism in the posterior cingulate cortex is also absent, while a connection between higher hydrolysis rate and APOE4 positivity exists (Kuhl et al., 1999; Eggers et al., 2006).

**Figure 7:**
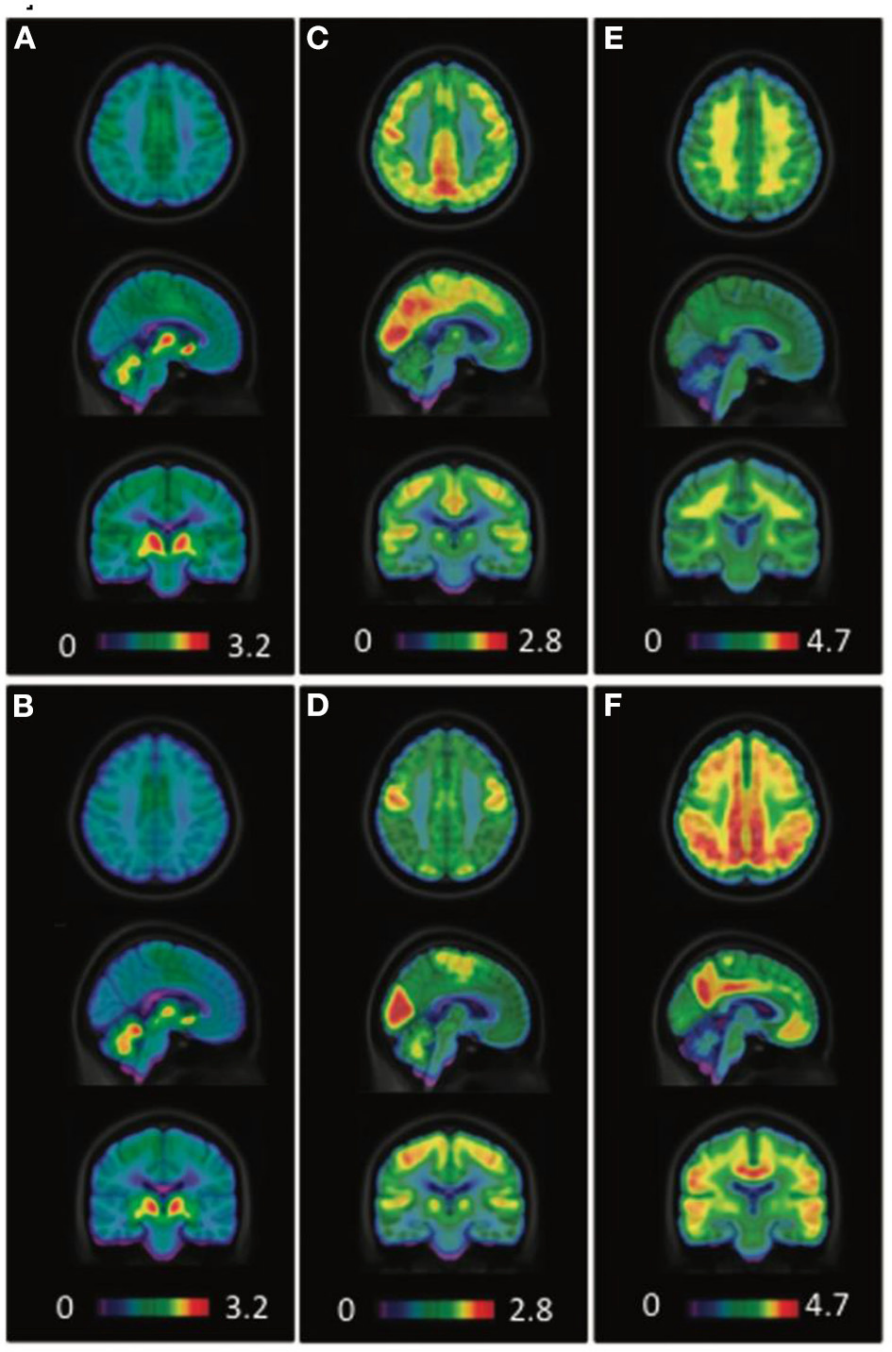
Averaged [^18^F]FEOBV VAChT PET **(A,B)**, [^18^F]FDG PET **(C,D)**, and [^18^F]NAV4694 Aβ PET **(E,F)** images of healthy controls **(A,C,E)** and AD patients **(B,D,F)**. PET images in control subjects revealed the greatest [^18^F]FEOBV uptake in brain areas known to be the most innervated by the cholinergic systems, including the striatum, thalamus, cerebral cortex as a whole, hippocampal area and cerebellum **(A)**. In AD patients, PET imaging revealed a significant reduction of [^18^F]FEOBV uptake in comparison with control subjects **(B)**. Distinct cerebral hypometabolism **(D)** and amyloid deposition **(F)** were seen in the AD patients, as compared to control subjects **(C,E)**. Courtesy from Aghourian et al. (2017).

The binding of novel VAChT tracer [^18^F]FEOBV is found to be lower in AD than in healthy controls in a recent multi-tracer study involving [^18^F]FDG and [^18^F]NAV4694 as well (Aghourian et al., 2017). [^18^F]FEOBV PET is shown to have higher sensitivity than [^18^F]FDG in discriminating AD from healthy controls (Figure 7). There is also a positive correlation of [^18^F]FEOBV binding to minimal mental state examination (MMSE) and Montreal cognitive assessment (MoCA). A more recent study attributed basal forebrain degeneration in AD to the loss of cortico-amygdalar cholinergic input, as demonstrated by [^18^F]FEOBV binding decline (Schmitz et al., 2018).

Nicotinic AChR binding reduction is not only lower in AD and MCI subjects than in cognitively-intact volunteers, but also in MCI converters to AD dementia than in non-converters (Nordberg et al., 1990, 1995; Kendziorra et al., 2011). Binding quantitation of the AD-affected brain regions is significantly responsible for their coupling cognitive functions (Ellis et al., 2008; Sabri et al., 2008; Kendziorra et al., 2011; Okada et al., 2013). Frontal [^11^C]PIB retention is found to be inversely correlated with [^18^F]2-FA binding in the medial frontal cortex and nucleus basalis magnocellularis of AD patients (Maas et al., 2000). PET imaging with [^18^F]flubatine, a new-generation α_4_β_2_ tracer with more favorable kinetics, reveals receptor deficiency within the basal forebrain-cortical and septo-hippocampal cholinergic projections (Sabri et al., 2018). Episodic memory, working memory and executive functions are well-correlated with impairment of α_4_β_2_ nAChR in the corresponding cortices. Initial imaging studies with the α_7_ nAChR selective radioligand [^18^F]ASEM in healthy controls and MCI subjects indicated elevation of this receptor subtype with healthy aging, and in MCI, in a direction opposite of that for α_4_β_2_ nAChR in AD (Coughlin et al., 2018, 2019). A preliminary imaging study of M4 mAChR in AD revealed reduced uptake of [^11^C]MK-6884 primarily in the parietotemporal cortex in a pattern consistent with clinical symptom presentations and FDG hypometabolism (Masdeu et al., 2020).

### Discussion

PET imaging studies with tracers for both pre- and post-synaptic cholinergic targets have confirmed the abnormalities of cholinergic transmission *in vivo*, in AD, as manifested in altered levels of AChE, VAChT, and α_4_β_2_ and α_7_ nAChR's, and their correlations with Aβ deposition and cognitive functions. The α_7_ nAChR is a target gaining increasing focus in recent years, due to its newly discovered participation in preventing amyloid toxicity and tau hyper-phosphorylation, in increasing synaptic strength and stability, and in modulating neuroinflammation by acting on non-neuronal cells (Wang et al., 2003; Conejero-Goldberg et al., 2008; Halff et al., 2014; de Oliveira et al., 2016; Maurer and Williams, 2017; Gamage et al., 2020). Renewed interests have also been seen in the mAChR, especially the M1 and M4 subtypes, for therapeutic development for AD (Levey, 1996; Schliebs and Arendt, 2011; Melancon et al., 2013). Investigation of these additional targets with recently available PET tracers will lead to a more thorough understanding of cholinergic involvement in AD etiology and progression.

## PET Tracers for Imaging Synaptic Density

### Overview

The total number of synapses in the neocortices is approximately 164 × 10^12^ (Tang et al., 2001). Synapses are critical for neurotransmission in neuron-neuron interaction, the deficiency of which would result in neuronal dysfunction and consequent occurrence of neuropsychiatric symptoms including amnesia, apathy and executive dysfunction. Loss of synapses has long been regarded as a pathologic hallmark of AD (Scheff et al., 1990; Terry et al., 1991; Scheff and Price, 2006) and correlates strongly with cognitive impairment (Hamos et al., 1989; DeKosky and Scheff, 1990; Terry et al., 1991; DeKosky et al., 1996; Robinson et al., 2014). Synaptic density reductions are seen in the neocortex and limbic system in MCI, a prodromal form of AD and other dementias (Masliah et al., 1994, 2001; Pham et al., 2010). Accumulation of toxic Aβ oligomers is believed to lead to the loss of synapses and presynaptic proteins in MCI patients (Pham et al., 2010; Wei et al., 2010; Beeri et al., 2012; Robinson et al., 2014). More recent research also suggests an emerging role for tau mediated toxicity at the synapses (Pooler et al., 2014; Wang and Mandelkow, 2016). Thus PET imaging of the synapses provides a tool for direct visualization of synaptic density loss along the AD pathogenesis and progression pathway.

### Tracer Development

**Figure 8:**
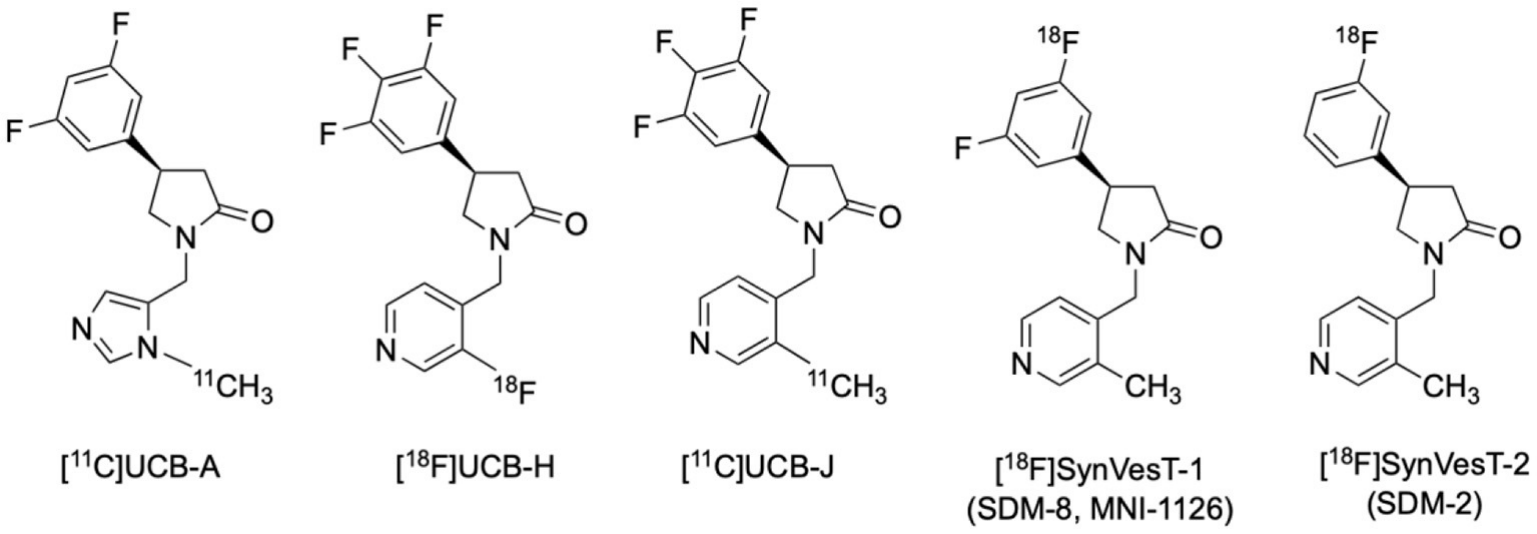
Structures of representative PET radiotracers for SV2A.

Synaptic vesicle glycoprotein 2 (SV2) is one of the synaptic proteins highly expressed on the presynaptic membrane. Among the 3 forms of SV2, only SV2A is extensively expressed in glutamatergic and GABAergic neurons throughout the CNS (Dong et al., 2006). Traditionally, synapses can only be observed by electron microscopic and immunohistochemical examinations in biopsy and autopsy tissue samples. Recently a series of novel PET radiotracers selectively targeting SV2A have been developed (Figure 8) (Mercier et al., 2014; Estrada et al., 2016; Nabulsi et al., 2016; Becker et al., 2017; Li et al., 2019; Cai et al., 2020a), enabling non-invasive observation and quantification of synaptic density in humans (Finnema et al., 2016; Bahri et al., 2017; Cai et al., 2020b; Naganawa et al., 2020b).

### Imaging Research Findings and Clinical Relevance

A recent human SV2A PET study using [^11^C]UCB-J in cohorts of MCI/AD patients and age-matched cognitively intact elderly subjects revealed significant reduction of synaptic density in the hippocampus and entorhinal cortex (44 and 27%, respectively) (Chen et al., 2018). Moreover, statistically significant correlations were found between hippocampal synaptic density and episodic memory (Logical Memory II and Rey Auditory Verbal Learning Test, *R* = 0.56, *P* = 0.01) and global function (Clinical Dementia Rating Sum of Boxes, *R* = −0.61, *P* = 0.003) (Chen et al., 2018). These findings suggest that [^11^C]UCB-J can capture the early pathological alterations in the hippocampus and entorhinal cortices, brain structures not readily analyzed in FDG PET compared to neocortices due to attenuated sensitivity introduced by size-related partial volume effect despite their early involvement in AD's Braak Staging (Braak and Braak, 1997).

The loss of synaptic density in the hippocampus of AD patients was replicated in a study using another SV2A radiotracer [^18^F]UCB-H, in which a 11 ~ 18% reductions of synaptic density in the basal forebrain and anterior/dorsomedial thalamus were found to be correlated with cognitive alterations (Bastin et al., 2020; Mecca et al., 2020). In a more recent [^11^C]UCB-J study with a larger cohort of 34 AD and MCI patients, the significant loss of synaptic density measured by PET in the hippocampus and entorhinal cortex followed by parahippocampal cortex, amygdala, lateral temporal cortex, pre-frontal cortex, posterior cingulate cortex/precuneus, lateral parietal cortex, and pericentral cortex was found to be generally correlated with atrophy measured by structural MRI which is in line with autopsy reports, and was more significantly correlated with CDR-SB (*r* = −0.54, *P* = 0.00003) and episodic memory (*r* = 0.56, *P* = 0.00001) than in the smaller sample reported earlier (de Wilde et al., 2016; Chen et al., 2018; Mecca et al., 2020). The relationship between cognitive performances and medial temporal (especially hippocampus and parahippocampal gyrus) synaptic density loss in aMCI was strengthened by a mutually correlated regional tau accumulation, as was revealed by a multi-tracer PET study using [^18^F]MK-6240 and [^11^C]UCB-J (Vanhaute et al., 2020).

### Discussion

SV2A PET has shown great promise as a biomarker for the non-invasive detection of synaptic density, which can serve as a useful tool for studying synaptopathies in neurodegenerative and psychiatric disorders including AD (Cai et al., 2019; Heurling et al., 2019). Due to the ubiquitous distribution of synaptic vesicles throughout the CNS, a highly selective molecular probe such as SV2A PET tracer can spot the subtle alterations of synaptic density both quantitatively and topographically.

**Figure 9:**
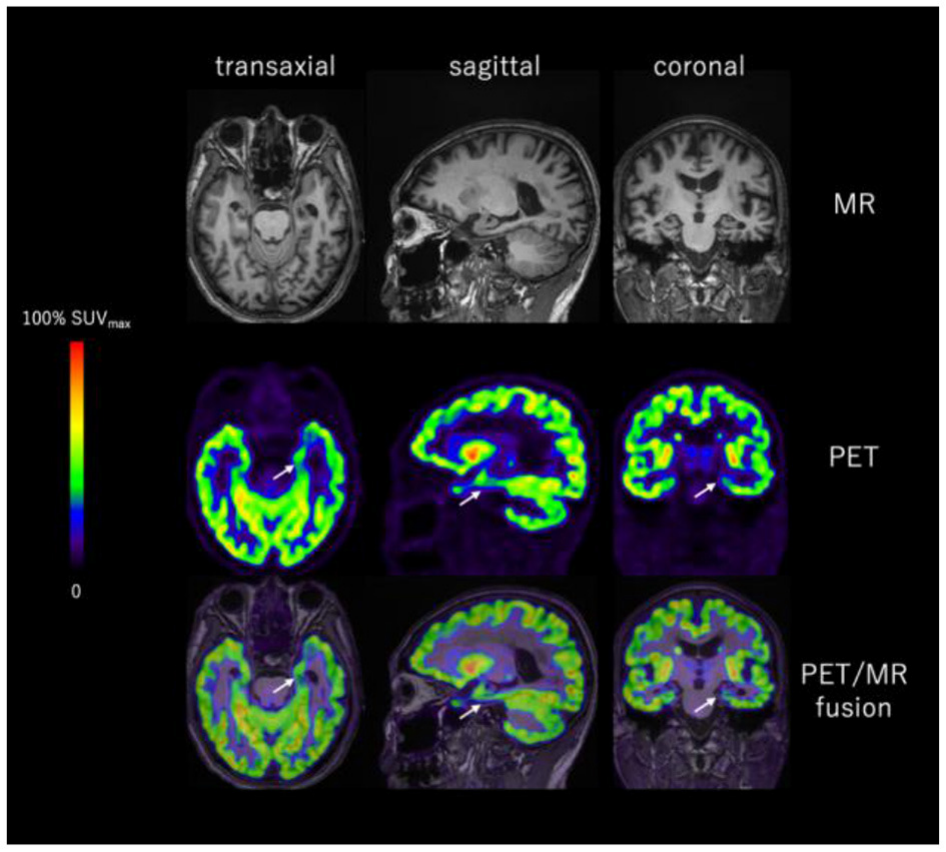
Representative images of T1WI MRI and [^18^F]SynVesT-1 PET of a [^18^F]Florbetapir positive AD patient. SV2A binding is significantly reduced in the left hippocampus (white arrow). Images provided by PET Center, Huashan Hospital, Fudan University and Department of Nuclear Medicine, East Hospital, Tongji University.

It is anticipated that with the help of multi-target molecular imaging approaches, the correlation between synaptic loss and other pathophysiological factors related to AD can be further elucidated. For instance, the relationship between pathological amyloid and tau protein aggregations, neuronal glucose utility impairment, dysfunctions in various neurotransmitter systems and synaptic loss would be better understood. Since pathological synaptic alterations emerge in the early stage of AD and continue throughout its course, many unanswered questions and controversial issues, e.g., sequence on the emergence of pathologic and physiological biomarkers, energetic, and neurotransmitter system abnormalities, and their relative impacts on disease progression, would be hopefully settled. It is perceivable that SV2A PET has the potential in AD early detection, differential diagnosis and conversion prediction of the prodromal stage subjects. Many studies using SV2A PET imaging in AD and other neurodegenerative diseases are ongoing, and results are emerging rapidly in publications. The introduction of additional ^18^F-labled SV2A radiotracers with favorable kinetics and high specific binding profiles, such as [^18^F]SynVesT-1 (also known as [^18^F]SDM-8 and [^18^F]MNI-1126) and [^18^F]SynVesT-2 (also known as [^18^F]SDM-2), should facilitate these studies since they are suitable for central production and distribution for use in off-site imaging facilities (Figures 8, 9). Further, PET imaging with these SV2A radiotracers will be useful as endpoint measure in AD drug clinical trials (e.g. NCT03493282: Effect of CT1812 treatment on brain synaptic density).

## Summary and Concluding Remarks

Alzheimer's disease, characterized by impairments of cognitive functions and memory loss, is an extremely complex neurodegenerative disorder involving multiple pathophysiological processes, from loss of synapses and neurons, to neuroinflammation, to deposition of Aβ plaques and neurofibrillary tau tangles. Early diagnosis is the key to effectively treat the disease before manifestation of clinical symptoms. The past two decades have witnessed the development of PET imaging agents targeting various pathologic and physiological biomarkers in AD which has greatly contributed to the investigation and diagnosis of this disease.

Radiotracers targeting pathologic hallmarks of AD such as Aβ and tau proteins have allowed the differentiation of AD and non-AD dementia, the tracking of pathological protein burden over the temporal course of the disease, the stratification of patients for clinical trials, and the monitoring of biological effects of therapeutic agents. Nonetheless, there remained a few unanswered questions to be further studied concerning the relationship between these targets and the etiology of AD.

Pathophysiological alterations such as neuroinflammation as well as neurotransmitter and synaptic dysfunction are increasingly thought to commence from the earliest stage of the pathological changes in AD, and PET radiotracers targeting these biomarkers may help to elucidate their roles in AD progression in synergy with the core pathologies.

Measurement of synaptic density through PET imaging of the synaptic biomarker SV2A opens a new avenue for the investigation of neurodegenerative diseases. Initial *in vivo* imaging results from prodromal AD and AD dementia cohorts were in excellent correlation with cognitive impairment. Preliminary results of healthy aging subjects also demonstrated the early involvement of synaptic density alterations and its relationship to tau aggregation, which is in concordance with autopsy neuropathological findings. SV2A PET imaging, now at its early discovery phase, is expected to provide further important information on synaptic protein alterations, along with other pathological changes, during the entire AD progression continuum, and contribute to the early detection of the disease.

PET imaging of biomarkers has played an important role in AD drug development. This trend will continue, as newer imaging biomarkers are increasingly incorporated into clinical trials of next-generation AD drugs as outcome measures. It is expected that applications of PET imaging biomarkers in drug clinical trials will continue to greatly facilitate the development of AD therapeutics.

The United States National Institute of Aging (NIA) and the Alzheimer's Association (AA) recently put forward a research framework for AD, i.e., the NIA-AA Research Framework, which is based on the current understanding of the AD continuum and grounded on a biomarker-based definition of AD (Jack et al., 2018). Biomarkers are grouped into three categories: Aβ deposition, pathologic tau, and neurodegeneration (ATN) based on the nature of the pathologic process that each measures. Biomarkers of Aβ plaques (labeled “A”) are represented by cortical Aβ plaque burden assessed by amyloid PET imaging or decreased CSF Aβ_42_. Biomarkers of fibrillar tau (labeled “T”) are elevated CSF phosphorylated tau (p-tau) and/or cortical tau tangles measured by tau PET imaging. Biomarkers of neurodegeneration or neuronal injury (labeled “N”] are based on CSF total tau (t-tau), hypometabolism as revealed by FDG PET imaging, and brain atrophy detected by MRI. It is evident that PET imaging targeting various biomarkers is an essential component of this ATN research framework, as it provides one biomarker measurement for each of the arms: amyloid PET for A, tau PET for T, and FDG PET for N. The recent emergence of synaptic PET imaging targeting SV2A may provide another biomarker for N, neurodegeneration that more closely tracks with the progression of the disease and cognitive impairment, and less sensitive to cofounding factors such as blood glucose level, stimulation and medication which affect FDG PET, a surrogate measure of synaptic/neuronal function (Herholz, 2008; Burns et al., 2013; Ishibashi et al., 2015). These advancements in the development of PET radiotracers for AD biomarkers have been made possible through the efforts of numerous researchers in the PET imaging field in the last two decades. Collectively, AD imaging studies with various radiotracers have greatly increased our knowledge on the etiology and progression of this complex neurodegenerative disorder, and facilitated the research and development of AD therapeutics. It is believed that PET imaging with effective tracers targeting various AD pathophysiological biomarkers will continue to advance our understanding of the disease, and hopefully provides a sensitive and definitive tool for early diagnosis and monitoring of treatment effect.

## Author Contributions

WB and YH drafted the manuscript. FX, CZ, and YG participated in revision of manuscript. All authors contributed to the article and approved the submitted version.

## Conflict of Interest

The authors declare that the research was conducted in the absence of any commercial or financial relationships that could be construed as a potential conflict of interest.
